# Management of Renal Cell Carcinoma With Supradiaphragmatic Inferior Vena Cava Thrombus Diagnosed During Acute COVID-19 Infection

**DOI:** 10.7759/cureus.55565

**Published:** 2024-03-05

**Authors:** Michael Leyderman, Ian M McElree, Kenneth G Nepple, Yousef Zakharia, Saum Ghodoussipour, Vignesh T Packiam

**Affiliations:** 1 Urology, Norton College of Medicine, SUNY Upstate Medical University, Syracuse, USA; 2 Urology, Carver College of Medicine, University of Iowa, Iowa City, USA; 3 Urology, University of Iowa Hospitals and Clinics, Iowa City, USA; 4 Internal Medicine - Hematology/Oncology, University of Iowa Hospitals and Clinics, Iowa City, USA; 5 Division of Urologic Oncology, Rutgers Cancer Institute of New Jersey, New Brunswick, USA

**Keywords:** covid-19, coronavirus, inferior vena cava thrombus, renal cell carcinoma, oncology, urology

## Abstract

Renal cell carcinoma (RCC) tends to undergo intravascular tumor growth along the renal vein, forming tumor thrombi that may extend into the inferior vena cava (IVC) or even the right atrium (Level IV). Managing such cases requires a multidisciplinary approach, especially in patients with acute coronavirus disease 2019 (COVID-19) infection, who face increased risks from surgical interventions. We present a case of RCC with Level IV thrombus and concurrent COVID-19 managed with systemic therapy. We also summarize current literature on treating RCC with IVC thrombus and COVID-19's impact on prognosis.

The patient was a 70-year-old female with incidental detection of a 9-cm right heterogeneous renal mass with a supradiaphragmatic tumor thrombus during COVID-19 infection. Due to ongoing pulmonary symptoms, systemic therapy with a combination of ipilimumab and nivolumab was initiated. After an excellent initial response, the patient continued systemic therapy, maintaining a necrotic response in the renal mass and tumor thrombus. The patient continues to tolerate systemic therapy well.

We report a rare case of RCC with Level IV tumor thrombus and synchronous acute COVID-19 infection. Our report depicts successful management utilizing systemic therapy with a combination of ipilimumab and nivolumab. The management of such cases necessitates a comprehensive, multidisciplinary approach, considering the risks associated with surgery in the context of recent COVID-19 infection. The case presentation and ensuing literature discussion of the dynamic landscape of RCC management highlights the need for more research to improve treatment plans and guide clinicians in handling such complex situations.

## Introduction

Renal cell carcinoma (RCC) is responsible for over 180,000 deaths worldwide, and accounts for 2.4% of all cancer diagnoses worldwide [1]. RCC with tumor thrombus extension occurs in 5-15% of RCC cases and requires prompt intervention [2]. Mayo Level IV thrombus, defined as a thrombus extending above the diaphragm or into the right atrium, occurs in 1% of cases and is particularly life-threatening, traditionally requiring urgent surgical management [3]. Novel combinations of systemic immunotherapies have promising efficacy for RCC but their role in the management of extensive tumor thrombus is unclear [4].

Coronavirus disease of 2019 (COVID-19) infection has been shown to increase peri-operative respiratory complications and mortality for patients undergoing surgery [5]. COVID-19 has also been shown to negatively impact mortality rates when operative interventions are performed in the acute phase [6, 7]. We present a case of a patient with synchronous diagnoses of RCC with Level IV inferior vena cava (IVC) thrombus and acute COVID-19 infection effectively managed with a combination of ipilimumab and nivolumab systemic therapy [8, 9]. We also discuss the current literature regarding IVC thrombus treatments and the clinical impact of COVID-19 in this setting.

## Case presentation

**Figure 1:**
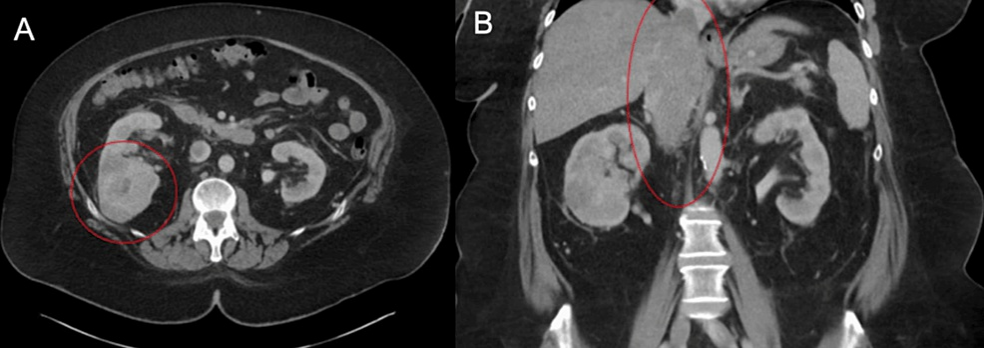
CT imaging of the abdomen and pelvis at initial presentation. (A) Axial view with 9 cm right renal mass. (B) Coronal view showing superior extent of occlusive thrombus above the diaphragm.

A 70-year-old female presented to the emergency room with shortness of breath and hypoxemia where she was diagnosed with COVID-19. A computed tomography (CT) angiogram of the chest demonstrated an incidental 9-cm right heterogeneous renal mass with an expansile tumor thrombus extending above the diaphragm and a 1.2-cm right adrenal nodule. The patient underwent a CT of the abdomen and pelvis with contrast which did not demonstrate any lymphadenopathy or metastatic disease. MRI of the abdomen with and without contrast showed that the IVC thrombus extended to the right atrium. There was also a bland nonocclusive infrarenal thrombus extending to below the aortic bifurcation (Figure 1). The patient was hospitalized for one week for acute respiratory failure and persistent hypoxemia secondary to COVID-19 infection and followed up with urology as an outpatient one week afterwards. At this time, she was experiencing persistent respiratory distress. A thorough risk stratification was conducted jointly by pulmonology and urology. Pulmonology consultation suggested postponing surgery for at least eight weeks. This delay would allow time for pulmonary recovery and rehabilitation, thereby facilitating a major multi-disciplinary surgery while also mitigating the heightened risk of COVID-19-associated mortality.

Due to the increased risk of surgery with recent COVID-19 infection, and the need for urgent management of the tumor thrombus, Medical Oncology was promptly consulted. Following a thorough risk analysis conducted in conjunction with urology, the potential for systemic therapy was carefully explored. A renal mass biopsy was obtained which demonstrated at least grade 3/4 clear cell RCC (ccRCC) based on the World Health Organization/International Society of Urological Pathologists (WHO/ISUP). Options were discussed and the patient consented to a clinical trial of lenvatinib and everolimus. Therapeutic anticoagulation with Xarelto was also initiated. The patient completed two months of pre-planned treatment with lenvatinib and everolimus, and after a good initial tumor response, developed adrenal insufficiency. Multidisciplinary discussion was held, and it was felt that the patient remained ineligible to tolerate major surgery, and thus, the patient was transitioned to further systemic therapy with ipilimumab and nivolumab.

**Figure 2:**
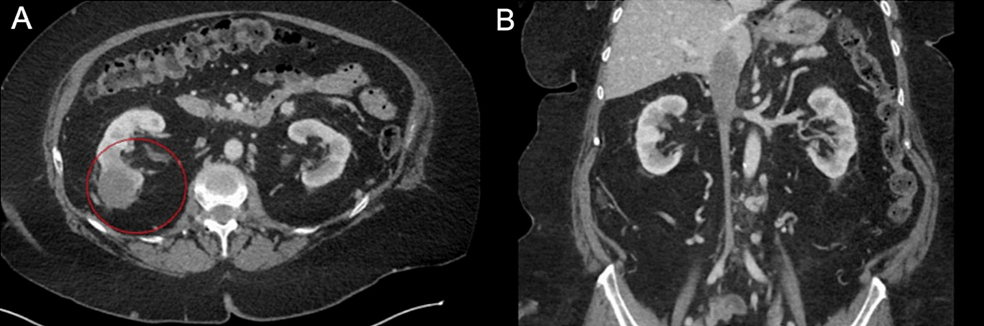
CT imaging of the abdomen and pelvis after one year of systemic therapy. (A) Axial view with significant necrotic shrinkage of primary renal mass. (B) Coronal view demonstrating necrotic shrinkage of thrombus below the diaphragm.

After four cycles of nivolumab and ipilimumab with standard dosing i.e. 3 mg per kilogram over a period of 60 minutes and 1 mg per kilogram over a period of 30 minutes, respectively, CT imaging showed an excellent necrotic response and a significant decrease in the size of the right renal mass and tumor thrombus (Figure 2). She was transitioned to single-agent nivolumab. Based on the clinical impression of the medical oncology and urologic teams, the patient had excellent tolerance to the systemic therapy with good functional status, appetite, and energy. Consolidative treatment options including surgery and radiation were discussed, but the patient elected to continue systemic therapy. Upon follow-up, the patient continued to tolerate the systemic therapy well.

## Discussion

There is considerable morbidity and mortality associated with nephrectomy with IVC thrombectomy, particularly as the superior aspect of the tumor thrombus extends above the diaphragm [10, 11]. Traditionally, treatment of RCC with tumor thrombi is done via radical nephrectomy with tumor thrombectomy, with the surgical approach becoming increasingly more complex as tumor thrombi expand upward into the IVC. Surgery for RCC and level IV thrombus is multi-disciplinary and typically involves cardiothoracic and hepatobiliary surgeons, as well as cardiopulmonary bypass [12]. This increases the risk of cardiopulmonary complications relative to other urologic operations. The primary etiologies of perioperative mortality include cardiac arrest, tumor embolization, venous thromboembolism, and hemorrhage. Late mortality may also result from renal failure and dialysis, and the need for additional operations, as shown by one study that performed risk stratification by tumor thrombus level [13].

Recent advancements in systemic therapy for RCC have transformed the treatment landscape for both metastatic and locally advanced cases. Notably, the application of these therapies has expanded to address large RCC-associated tumor thrombus, with the aim of mitigating the necessity for immediate surgical intervention. Dual therapy involving ipilimumab and nivolumab has demonstrated remarkable efficacy in managing metastatic RCC. The success of this approach is evident in its ability to substantially reduce the superior extent of level IV tumor thrombus, presenting a promising alternative to surgery above the diaphragm, in select cases [12].

In the context of locally advanced ccRCC, with or without IVC thrombi, emerging evidence advocates for the consideration of immune checkpoint inhibitors (ICIs) as a first-line treatment option, particularly when safety concerns impede surgical procedures. Existing case reports underscore the potential of systemic therapy in downstaging IVC thrombi, resulting in diminished tumor size and facilitating less invasive vascular surgery. Notably, this approach has shown promise in decreasing rates of complications associated with surgical interventions [13]. While there is limited medical literature on an immunotherapy-only approach, neoadjuvant treatments have proven successful in tumor debulking and enhancing surgical outcomes.

A noteworthy study by Master et al. in 2023 showcased the successful use of ipilimumab and nivolumab in nonmetastatic RCC with tumor thrombus in the neoadjuvant setting [14]. This intervention led to a decrease in tumor size, with no severe immune adverse events. However, it necessitated surgical thrombus extraction due to incomplete regression below the right atrium. Other studies have highlighted the neoadjuvant benefits of checkpoint inhibition, demonstrating their efficacy in downstaging tumors and the associated thrombus, as well as alleviating symptoms such as lower extremity edema and dyspnea [15, 16]. Overall, the administration of immune checkpoint inhibitors before surgery has shown promise in lowering perioperative morbidity and potentially enhancing postoperative recurrence-free and progression-free survival [4].

Several studies have found that a diagnosis of COVID-19 leads to a greater risk of complications and mortality after surgery [17, 18]. In the setting of COVID-19, thromboembolic complications can result in morbidity - patients with COVID-19 can exhibit increased D-dimer levels and a greater risk of thrombosis and in-hospital mortality [19]. Although surgical treatment has associated thromboembolic risks, immune checkpoint inhibitors can also result in an increased risk of thrombosis. Once cytotoxic T-lymphocyte associated protein 4 (CTLA-4) and programmed cell death protein 1 (PD-1) antibodies bind to their respective receptor on activated T cells, there are increased cytokines and neutrophil extracellular traps and an increase in endothelial inflammation and accelerated atherosclerosis which exacerbate vascular lesions [20]. The risk of venous thromboembolism increases 5-8% within six months of starting treatment and 10-15% within 12 months, while the risk of arterial thromboembolism increases 1-2% within 6-17 months [20]. There are additional risks in using immune checkpoint inhibitors in the setting of COVID-19. There is a possible overlap of COVID-related interstitial pneumonia and cytokine release syndrome with the pulmonary toxicity of ICIs [21]. Management therefore can also require careful consideration of the viral exposure profile of patients.

The role of consolidative surgery in RCC after excellent response to systemic therapy is unclear and controversial. The Cancer du Rein Metastatique Nephrectomie et Antiangiogéniques (CARMENA) study importantly did not show a benefit of cytoreductive nephrectomy (CN) after tyrosine kinase inhibition in intermediate-poor risk metastatic disease. However, recent studies have shown some benefits with CN when systemic therapy is administered utilizing newer agents [22, 23]. Other non-surgical approaches have also been utilized. Stereotactic body radiotherapy has shown activity for RCC and can be a well-tolerated localized therapy to combine with systemic therapy [24-26]. Radiofrequency ablation (RFA) is another less invasive technique that employs high-frequency electrical current to destroy tumor tissue and can offer shorter recovery times compared to surgery [27]. However, limitations include the risk of tissue charring and challenges in achieving adequate ablation margins, particularly in larger tumors which may require several ablation sessions [27]. Cryoablation exhibits promising results in the management of renal masses, especially those <3 cm, compared to invasive techniques like partial nephrectomy [28]. Although some studies discuss the synergy between immunotherapy and ablation techniques [29-30], prospective clinical trials are necessary to substantiate this clinical benefit. At this time, the decision between consolidative surgery, radiation, or ablation is made on a case-by-case basis, and the optimal management after excellent or suspected complete response is unknown.

## Conclusions

Management of RCC with tumor thrombus is increasingly complex due to comorbidities such as acute COVID-19 infection. Our report depicts the successful use of systemic therapy to manage a large renal mass and significant IVC thrombus in a patient with recent severe COVID-19 infection. The management of such cases necessitates a comprehensive and multidisciplinary approach, considering the risks associated with surgery in the context of recent COVID-19 infection. Combination immunotherapy (ipilimumab and nivolumab) in this instance proves promising over immediate surgery. The ramifications of COVID-19 on surgical outcomes and the potential risks linked to immune checkpoint inhibitors emphasize the importance of personalized and cautious management, underscoring the need for additional larger-scale studies to refine treatment approaches and effectively navigate the complexities of these situations.
